# The mitochondrial genome of *Lamellomphalus manusensis* Zhang & Zhang, 2017 (Gastropoda: Neomphalida) from deep-sea hydrothermal vent

**DOI:** 10.1080/23802359.2022.2073843

**Published:** 2022-05-10

**Authors:** Shuqian Zhang, Suping Zhang

**Affiliations:** a Laboratory of Marine Organism Taxonomy and Phylogeny, Institute of Oceanology, Chinese Academy of Sciences, Qingdao, China; b Center for Ocean Mega-Science, Chinese Academy of Sciences, Qingdao, China

**Keywords:** Neomphalidae, Peltospiridae, mitogenome, phylogenetics

## Abstract

We report the nearly complete mitochondrial genome sequence of *Lamellomphalus manusensis* Zhang and Zhang 2017, a deep-sea snail inhabiting hydrothermal vent. The mitogenome is 15,990 bp in length, has a base composition of A (35.7%), T (33.5%), C (15.4%) and G (15.4%), and contains 13 protein-coding genes, 2 ribosomal RNA genes, and 22 tRNA genes. Phylogenetic analyses show that the family Peltospiridae is not monophyletic, suggesting that its genera need to be redefined.

Neomphalida (Warén and Bouchet 1993) are a group of gastropods inhabiting deep sea hydrothermal vents and sunken wood (Zhang and Zhang 2017). Since its discovery, phylogenetic position of this group was uncertain and had been placed as the sister group of Vetigastropoda (Ponder and Lindberg 1997; Warén et al. 2003), within the Vetigastropoda (Aktipis and Giribet 2012) or closest to Cocculinoidea (Aktipis and Giribet 2012). Until recent years, based on mitochondrial genomes, Uribe et al. (2016) recovered Neomphalida as a distinct lineage sister to other clades including Vetigastropoda and Neritimorpha + Caenogastropoda. In the present study, we report the nearly complete mitogenome of *Lamellomphalus manusensis* Zhang and Zhang 2017, the first representative of the family Neomphalidae for which such data is available, to provide more mitochondrial genome data for further phylogenetic studies.

The specimen was collected from a hydrothermal vent of the Manus Back-Arc Basin at depth of 1,740 m (3°44′02″S 151°40′39″ E), and have been deposited at Marine Biology Museum of Chinese Academy of Sciences (voucher number: M060, Shuqian Zhang, zsqtaxon@qdio.ac.cn). Research operations in the Manus Back-Arc Basin were carried out under permission from the government of Papua New Guinea, through a formal diplomatic declaration. Genomic DNA was extracted using OMEGA Mollusk DNA Kit. Paired-end sequencing (2 × 150 bp) was performed in an Illumina NextSeq sequencer. Quality control of the raw data was performed using Trimmomatic (Bolger et al. 2014) by removing adapters, duplicated sequences, reads with a quality score below 20 (Q < 20), and reads containing a percentage of uncalled bases (“N” characters) equal to or greater than 10%. Around 0.87% of raw reads (576,181 out of 66,300,344) were assembled into contigs using ABySS v2.0.2 (http://www.bcgsc.ca/platform/bioinfo/software/abyss) to produce a single mitogenome with an average 36.03× coverage. Subsequently, we annotated the assembled mitochondrial genome via DOGMA (Wyman et al. 2004). The exact initiation and termination codon positions of the protein-coding genes and the boundaries of the ribosomal RNA (rRNA) genes were manually verified. The mitogenome of *Lamellomphalus manusensis* Zhang and Zhang 2017 was 15,990 bp in length (Accession No. OK552681), and is composed of 35.7% A, 33.5% T, 15.4% C and 15.4% G. It typically contains 13 protein-coding, 2 ribosomal RNA, and 22 tRNA genes. The mitogenome had 30 small non-coding regions ranging from 1 to 59 bp. The only large non-coding region was 612 bp in length, which is likely the control region.

Concatenated amino-acid sequences of all 13 PCGs and two rRNA genes were used in Bayesian inference and maximum likelihood analyses. Reconstructed phylogeny showed that *Lamellomphalus manusensis* Zhang and Zhang 2017 and the other two species of Neomphalida formed a fully supported clade that is closest to a cocculiniformia species (Figure 1). The result supports the Neomphalida as a distinct lineage sister to other clades including Vetigastropoda and Neritimorpha + Caenogastropoda, as suggested by Aktipis and Giribet (2012) and Uribe et al. (2016). Within Neomphalida, *Lamellomphalus manusensis* Zhang and Zhang 2017 together with *Chrysomallon squamiferum* Chen, Linse, Copley & Rogers, 2015 (Peltospiridae) form a highly supported clade (PP = 1, BP = 88) sister to *Peltospira smaragdina* Warén and Bouchet 2001 (Peltospiridae), suggesting that Peltospiridae is nonmonophyletic. The classification of family Peltospiridae thus should be redefined.

**Figure 1.**
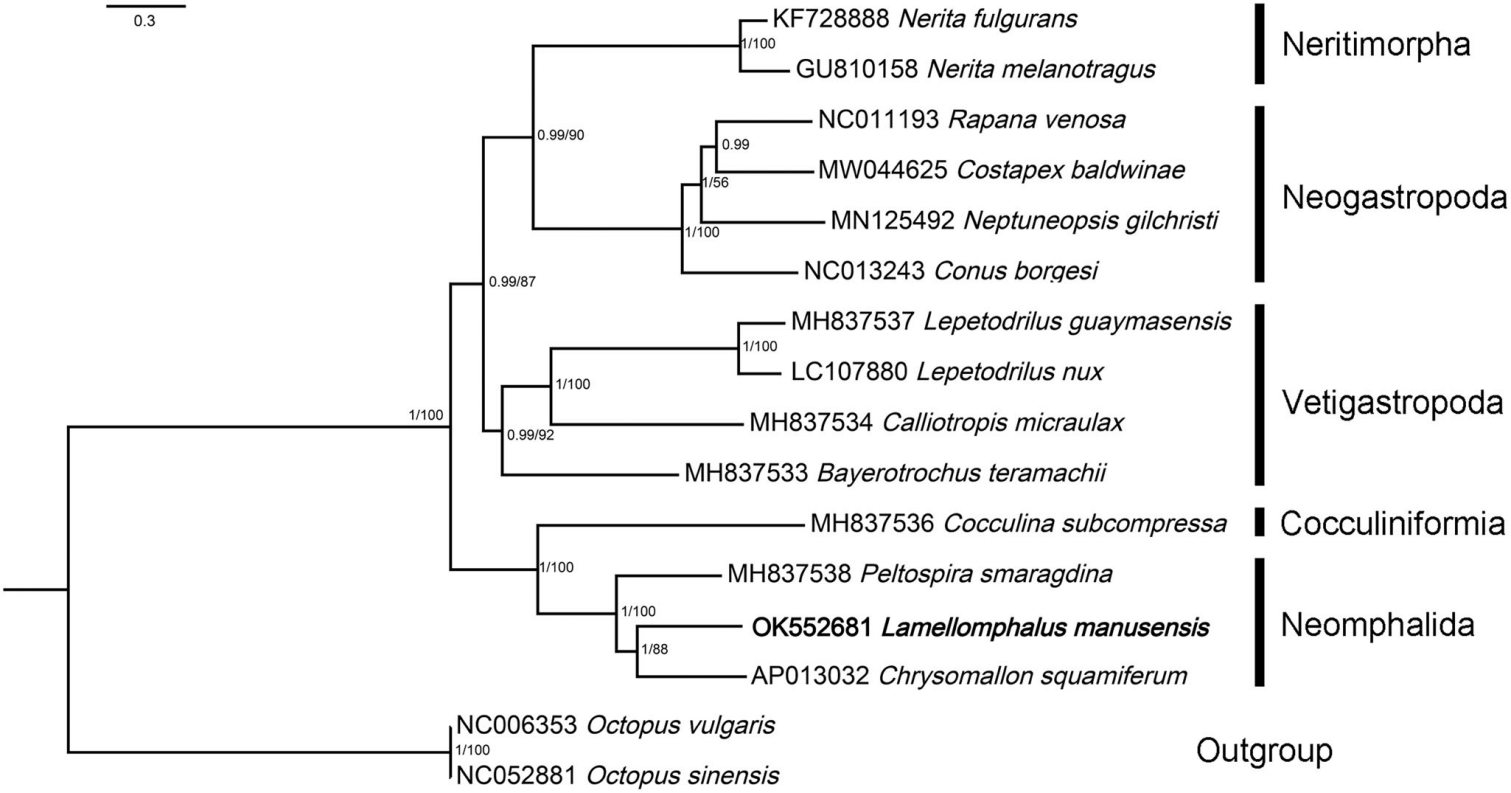
Relationships of *Lamellomphalus manusensis* to representive members of the Gastropoda. Nucleotide sequences of all protein-coding genes and ribosomal genes were individually aligned using MAFFT v. 7 (Katoh et al. 2019), ambiguous positions removed using GBlocks (Talavera and Castresana 2007), then concatenated, leading to alignments with 10,979 nucleotide positions for protein-coding genes and 1982 positions for the rRNA genes. ModelFinder (Kalyaanamoorthy et al. 2017) was used to select the best-fit partition model (Edge-unlinked) using BIC criterion. Maximum likelihood phylogenies were inferred using IQ-TREE (Nguyen et al. 2015) under the GTR + R4 + F model for 10,000 ultrafast (Minh et al. 2013) bootstraps, as well as the Shimodaira–Hasegawa–like approximate likelihood-ratio test (Guindon et al. 2010). Bayesian Inference phylogenies were inferred using MrBayes 3.2.6 (Ronquist et al. 2012) under partition model (2 parallel runs, 2,000,000 generations), in which the initial 25% of sampled data were discarded as burn-in. Branch support shown as maximum-likelihood bootstrap values (when ≥50)/Bayesian posterior probability (when ≥0.8).

## Data Availability

The genome sequence data that support the findings of this study are openly available in GenBank of NCBI at [https://www.ncbi.nlm.nih.gov] under the accession no. OK552681. The associated BioProject, SRA, and Bio-Sample numbers are PRJNA798177, SRR17651057, and SAMN25049529, respectively.

## References

[CIT0001] Aktipis SW, Giribet G. 2012. Testing relationships among the vetigastropod taxa: a molecular approach. J Mollus Stud. 78(1):825–27.

[CIT0002] Bolger AM, Lohse M, Usadel B. 2014. Trimmomatic: a flexible trimmer for Illumina sequence data. Bioinformatics. 30(15):2114–2120. 24695404 10.1093/bioinformatics/btu170 PMC4103590

[CIT0003] Guindon S, Dufayard JF, Lefort V, Anisimova M, Hordijk W, Gascuel O. 2010. New algorithms and methods to estimate maximum-likelihood phylogenies: assessing the performance of PhyML 3.0. Syst Biol. 59(3):307–321. 20525638 10.1093/sysbio/syq010

[CIT0004] Kalyaanamoorthy S, Minh BQ, Wong TKF, von Haeseler A, Jermiin LS. 2017. ModelFinder: fast model selection for accurate phylogenetic estimates. Nat Methods. 14(6):587–589. 28481363 10.1038/nmeth.4285 PMC5453245

[CIT0005] Katoh K, Rozewicki J, Yamada KD. 2019. MAFFT online service: multiple sequence alignment, interactive sequence choice and visualization. Brief Bioinform. 20(4):1160–1166. 28968734 10.1093/bib/bbx108 PMC6781576

[CIT0006] Minh BQ, Nguyen MA, von Haeseler A. 2013. Ultrafast approximation for phylogenetic bootstrap. Mol Biol Evol. 30(5):1188–1195. 23418397 10.1093/molbev/mst024 PMC3670741

[CIT0007] Nguyen LT, Schmidt HA, von Haeseler A, Minh BQ. 2015. IQ-TREE: a fast and effective stochastic algorithm for estimating maximum-likelihood phylogenies. Mol Biol Evol. 32(1):268–274. 25371430 10.1093/molbev/msu300 PMC4271533

[CIT0008] Ponder WF, Lindberg DR. 1997. Towards a phylogeny of gastropod molluscs: an analysis using morphological characters. Zool J Linn Soc. 119(2):83–265.

[CIT0009] Ronquist F, Teslenko M, van der Mark P, Ayres DL, Darling A, Höhna S, Larget B, Liu L, Suchard MA, Huelsenbeck JP. 2012. MrBayes 3.2: efficient Bayesian phylogenetic inference and model choice across a large model space. Syst Biol. 61(3):539–542. 22357727 10.1093/sysbio/sys029 PMC3329765

[CIT0010] Talavera G, Castresana J. 2007. Improvement of phylogenies after removing divergent and ambiguously aligned blocks from protein sequence alignments. Syst Biol. 56(4):564–577. 17654362 10.1080/10635150701472164

[CIT0011] Uribe JE, Kano Y, Templado J, Zardoya R. 2016. Mitogenomics of vetigastropoda: insights into the evolution of pallial symmetry. Zool Scr. 45(2):145–159.

[CIT0012] Warén A, Bengtson S, Goffredi SK, van Dover CL. 2003. A hot-vent gastropod with iron sulfide dermal sclerites. Science. 302(5647):1007. 14605361 10.1126/science.1087696

[CIT0013] Wyman SK, Jansen RK, Boore JL. 2004. Automatic annotation of organellar genomes with DOGMA. Bioinformatics. 20(17):3252–3255. 15180927 10.1093/bioinformatics/bth352

[CIT0014] Zhang SQ, Zhang SP. 2017. A new genus and species of Neomphalidae from a hydrothermal vent of the Manus Back-Arc Basin, western Pacifific (Gastropoda: Neomphalina). Nautilus. 131:76–86.

